# Penalized canonical correlation analysis to quantify the association between gene expression and DNA markers

**DOI:** 10.1186/1753-6561-1-s1-s122

**Published:** 2007-12-18

**Authors:** Sandra Waaijenborg, Aeilko H Zwinderman

**Affiliations:** 1 Department of Clinical Epidemiology, Biostatistics and Bioinformatics, Academic Medical Center, P.O. Box 22700, 1100 DE Amsterdam, The Netherlands

## Abstract

Inter-individual variation in gene expression levels can arise as an effect of variation in DNA markers. When associating multiple gene expression variables with multiple DNA marker variables, multivariate techniques, such as canonical correlation analysis, should be used to deal with the effect of co-regulating genes. We adapted the elastic net, a penalized approach proposed for variable selection in regression context, to canonical correlation analysis. The number of variables within each canonical component could be greatly reduced without too much loss of information, so the canonical components become easier to interpret. Another advantage is that it groups co-regulating genes, so that they end up in the same canonical components. Furthermore, our adaptation works well in situations where the number of variables greatly exceeds the number of subjects.

## Background

Inter-individual variation in gene expression is due to differences in experimental, environmental, and biological factors. Many authors have related expression of single genes to variation of a single DNA marker, or several markers, mostly in the same gene. Although many details are still unknown, molecular research has shown that expression of genes is regulated by the expression of many other genes in a sometimes highly complex network. This means that expression of genes should not be analyzed separately, and if the association of gene expression with DNA markers is estimated, this should be done jointly.

Several multivariate techniques are available to estimate the relationship of a set of gene expression variables with a set of DNA marker variables. Most are based on principal components analysis, singular value decomposition, partial least squares, or variants thereof. One of the aims of these techniques is to explain the variation of many genes by a much smaller set of components that are sometimes called latent genes; these may coincide with regulatory networks. These components are weighted combinations of the original gene expression variables or the DNA marker variables, and these weights are inspected to interpret the components/latent genes. Interpretation is easier if many of these weights are zero, or near zero, and only a few non-zero. Rotation of the components is used for that, but this is often not successful, and instead penalized methods have been suggested.

In this paper we describe the use of a newly developed canonical correlation analysis (CCA) to estimate the association between gene expression variables and DNA marker variables, in which we employed the elastic net [1] to simplify interpretation of the CCA components. We will analyze the Centre d'Etude du Polymorphisme Humain (CEPH) expression data of the Genetic Analysis Workshop 15 as an illustration of our method.

## Methods

### Data

Data on 14 three-generation CEPH families, each consisting of four grandparents, two parents and seven to eight offspring, were provided by the Genetic Analysis Workshop 15. Gene expression levels in lymphoblastoid cells of these 194 subjects were obtained using the Affymetrix Human Focus Arrays that contain probes for 8500 transcripts. The data set contains 3554 of the 8500 genes, for which Morley et al. [2] found that the variation in expression level among individuals was higher than between replicates on the same individual. Furthermore, the genotypes of 2882 autosomal and X-linked single-nucleotide polymorphisms (SNPs) were provided.

Missing SNPs were imputed based on the SNP data of relatives or if incomplete, based on related and unrelated subjects. SNPs with a percentage of missing data exceeding 20% (187 monomorphic and 24 polymorphic), were discarded from further analysis, as were the 95 SNPs which had a mutation rate of less than 5% (homo- and heterozygote). In the SNPs with less than 5% homozygote mutations, the hetero- and homozygote mutants were combined into one category (958 SNPs). For each SNP variable two dummies were created, using the LogicFS library in R. The first dummy variable was coded 0 for wild type and 1 for the mutants. The second dummy variable was coded 0 for wild type and heterozygote mutant and 1 for homozygote mutant.

### Penalized canonical correlation analysis

Consider the standardized *n *× *p *matrix **Y**, containing *p *(gene expression) variables, and the standardized *n *× *q *matrix **X**, containing *q *(DNA marker) variables, obtained from *n *subjects. Canonical correlation analysis (CCA) looks for a linear combination of all the variables in one data set that correlates maximally with a linear combination of all the variables in the other data set. These linear combinations are the so-called CCA components ξ and ω, such that ξ = **Xv **and ω= **Yu**, with the weight vectors **u**^*T *^= (*u*_1_,..., *u*_*p*_) and **v**^*T *^= (*v*_1_,..., *v*_*q*_). The correlation between the CCA components is also known as the canonical correlation.

Via the two-block Mode B of Wold's original partial least-squares algorithm, the weight vectors can be estimated [3]. This algorithm is an iterative process that begins by calculating an initial pair of CCA components (ξ and ω) based on an initial guess of the weight vectors (**v **and **u**). The objective is to maximize the correlation between these two CCA components, therefore ξ is regressed on **Y **and ω is regressed on **X **in order to obtain a new set of estimated weights. This process is repeated until the weights converge.

However, problems arise in the regression step due to multicollinearity and overfitting. Furthermore, all the original variables are contained in the CCA components, so interpretation is difficult. Zou and Hastie [1] proposed the elastic net to overcome these problems. It is based on the following properties 1) variable selection is built into the procedure, 2) the procedure is not limited by the fact that the number of variables greatly exceeds the number of subjects, and 3) strongly correlated variables are in or out of the model together. The elastic net combines the advantages of the lasso [4] (built in variable selection) and ridge regression (grouping effect). We adapted the elastic net to Wold's algorithm, obtaining the following algorithm:

1. Set *k *← 0.

2. Assign arbitrary starting values ${\hat{v}}^{\left(0\right)}$ and ${\hat{u}}^{\left(0\right)}$. For instance, set ${\hat{v}}^{\left(0\right)}\leftarrow \frac{1}{q}1$ and ${\hat{u}}_{r}^{\left(0\right)}\leftarrow \frac{1}{p}1$. And normalize ${\hat{v}}^{\left(0\right)}$ and ${\hat{u}}^{\left(0\right)}$.

3. Estimate ξ, ω, **v **and **u **iteratively, as followed

a. *k *← *k *+ 1.

b. ${\hat{\xi }}^{k}\leftarrow X{\hat{v}}^{\left(k-1\right)}$ and ${\hat{\omega }}^{k}\leftarrow Y{\hat{u}}^{\left(k-1\right)}$.

c. Compute ${\hat{v}}^{\left(k\right)}$ and ${\hat{u}}^{\left(k\right)}$ by performing multiple regression using the elastic net.

$$\begin{array}{c} {\hat{v}}^{\left(k\right)}=\left(1+\lambda_{2X}\right)\arg\underset{v^{\left(k\right)}}{\min}|{\hat{\omega }}^{k}-Xv^{\left(k\right)}{|}^{2}+\lambda_{2X}\sum_{j=1}^{q}v_{j}^{(k)2}+\lambda_{1X}\sum_{j=1}^{q}\left|v_{j}^{\left(k\right)}\right|\\ {\hat{u}}^{\left(k\right)}=\left(1+\lambda_{2Y}\right)\arg\underset{u^{\left(k\right)}}{\min}|{\hat{\xi }}^{k}-Yu^{\left(k\right)}{|}^{2}+\lambda_{2Y}\sum_{j=1}^{p}u_{j}^{(k)2}+\lambda_{1Y}\sum_{j=1}^{p}\left|u_{j}^{\left(k\right)}\right| \end{array}$$

d. Normalize ${\hat{v}}^{\left(k\right)}$ and ${\hat{u}}^{\left(k\right)}$. Repeat until ${\hat{v}}^{\left(k\right)}$ and ${\hat{u}}^{\left(k\right)}$ have converged (a difference of less than 10^-3 ^with the preceding step).

Hereafter, the residual matrices of **Y **and **X **are determined and the algorithm can be repeated to obtain the next sets of CCA-components. This process can be repeated until the residual matrices contain no further information.

The penalty parameters *λ*_1 _and *λ*_2 _come from the lasso and the ridge penalty, respectively. The (1 + *λ*_2_) scaling factor is built in, to prevent a double shrinkage. By introducing this scaling factor, the shrinkage effect of the ridge is eliminated while maintaining the grouping effect of the ridge and the shrinkage effect of the lasso. The algorithm is highly depending on the choices of *λ*_1 _and *λ*_2_. Methods to determine their optimal values are discussed in the following sections.

### Univariate soft-thresholding

The optimal values of *λ*_1 _and *λ*_2 _are determined by cross-validation (see next section), but in cases in which the computation time is tremendously increased due to the high number of variables, as in the case of microarray data, it might be desirable to simplify the computations. As Zou and Hastie [1] proposed, we can set *λ*_2*X *_→ ∞ and *λ*_2*Y *_→ ∞, in order to simplify the calculation. This is called univariate soft-thresholding. Although it ignores the dependence between variables within the same set, the de-correlation effect from the ridge penalty is maintained. It has shown to be quite promising [1].

Performing univariate soft-thresholding, Step 3(c) reduces to computing ${\hat{v}}^{\left(k\right)}$ and ${\hat{u}}^{\left(k\right)}$ by

$$\begin{array}{cc} {\hat{v}}_{i}^{\left(k\right)}={\left(\left|{\hat{\omega }}^{kT}X_{i}\right|-\frac{\lambda_{1X}}{2}\right)}_{+}sign\left({\hat{\omega }}^{kT}X_{i}\right) & i=1,\ldots ,q\\ {\hat{u}}_{i}^{\left(k\right)}={\left(\left|{\hat{\xi }}^{kT}Y_{i}\right|-\frac{\lambda_{1Y}}{2}\right)}_{+}sign\left({\hat{\xi }}^{kT}Y_{i}\right) & i=1,\ldots ,p. \end{array}$$

From this formula, we see that now only the optimal lasso penalty has to be chosen. Lasso shrinks weights to zero; the larger the lasso penalty, the smaller the number of weights that receive a non-zero value. The magnitude of the lasso penalty determines how many weights become non-zero, and thus how many variables are maintained in the CCA-components. Following Zou and Hastie [1], we turned this process around and determined the number of variables we would like to give a non-zero weight.

### Penalty parameter optimization

The optimal number of variables within each CCA component is determined by *k*-fold cross-validation. The data set is divided into *k *subsets (based on subjects), of which *k*-1 subsets form the training set and the remaining subset forms the test set. The weight vectors **u **and **v **are estimated by the training set and the mean squared prediction error is determined by the test set. This is repeated *k *times, such that each subset has functioned as a test set.

The mean squared prediction error is defined by

$$MSPE={\frac{\sum_{j=1}^{N}\left|x_{j}{\hat{v}}^{-k(j)}-\rho^{-k(j)}y_{j}{\hat{u}}^{-k(j)}\right|}{N}}^{2},$$

with *ρ*^-*k*(*j*) ^the canonical correlation, and ${\hat{v}}^{-k(j)}$ and ${\hat{u}}^{-k(j)}$ the weight vectors estimated by the training set in which subject *j *was deleted. The MSPE is determined for CCA components with differing numbers of variables. The CCA component pair with the lowest MSPE contains the optimal number of variables.

## Results and discussion

### Data exploration

We obtained data set **Y**, containing 3554 gene expression variables, and data set **X**, containing 4194 SNP dummy variables from 194 subjects. Principal component analysis was used to explore the gene expression variables. It revealed a presence of gender effect and a separation between adults and children. In contrast with what we expected, there was no family effect (results not shown). To get rid of these gender and generation affects, we performed linear regression analysis of each gene expression variable on gender and generation (children versus parents and grandparents).

Intraset correlation of the gene expression variables varied between -0.82 and 0.94, indicating the presence of co-regulating genes in the data set. Interset correlation of single gene expression variables with single SNP dummy variables varied between -0.56 and 0.46, so the dummy variables of single SNPs did show some effect on the gene expression levels.

### CCA component optimization

**Figure 1:**
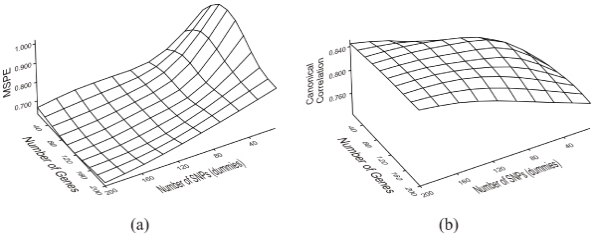
**Effect of the number of variables on the MSPE andthe canonical correlation**. The effect of the number of variables within each CCA component pair on (a) the mean squared prediction error, and (b) the average canonical correlation.

We performed genome-wide penalized canonical correlation analysis to determine the CCA components. The effect of the number of variables within each CCA component on the mean squared prediction error (MSPE) is shown in Figure 1a. The decreasing trend in the MSPE continued when the number of variables in both CCA components increased. Figure 1b shows the effect of the number of variables on the average canonical correlation of the training sets; there was an increasing trend when the number of variables increased, but the increase was less steep as the number of variables approached 200.

Although the MSPE decreased as the number of variables within each CCA component increased, this decrease became smaller when more variables were added to the components. The increase in canonical correlation also became smaller. For example, when we compared a pair of CCA components containing 200 variables each and a pair of CCA components containing 50 variables each, the increase in MSPE was 0.15 (0.65 to 0.80) and the decrease in canonical correlation was 0.05 (0.85 to 0.80). A compromise between MSPE, the canonical correlation, and the desired number of variables within each CCA component can be made to derive the optimal number of variables within each CCA component.

### Findings

**Figure 2:**
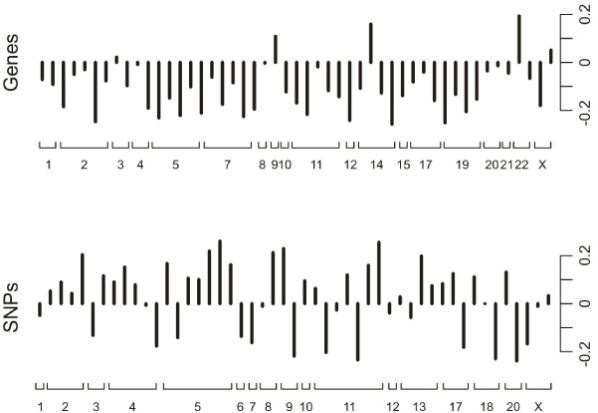
**Weights of the CCA components**. The weights of the CCA components containing the gene expression variables and the SNP dummy variables, ordered according to their chromosome location, obtained from a CCA component pair containing 50 variables each.

To keep the results interpretable, we decided to focus on the CCA components with 50 variables each. The first pair of CCA components was estimated using all 194 subjects, resulting in a canonical correlation of 0.81. The estimated weights are given in Figure 2. The variables within each CCA component were selected from nearly all chromosomes. The five genes with the largest weights were *HNRPAB*, *TACC3*, *SCHIP1*, *BAZ1B*, and *TUBG1*. And the five SNPs with the largest weights were rs131973, rs1351583, rs1862121, rs1010127, and rs616113.

**Figure 3:**
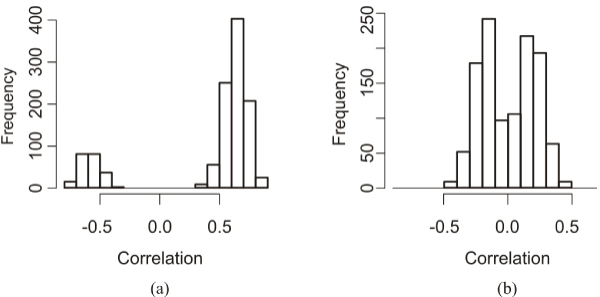
**Intra-CCA component correlation**. The distribution of the intra-CCA components correlations (a) of the gene expression variables and (b) the SNP dummy variables, obtained from a CCA component pair containing 50 variables each.

The intra-CCA component correlation of the variables is given in Figure 3. The absolute intra-CCA component correlation of the single gene expressions varied between 0.32 and 0.89, this indicated that co-regulating genes indeed ended up in the same model. The absolute intra-CCA component correlation of the single SNP dummies was somewhat lower. It varied between 0.0010 and 0.89. The inter-CCA component correlation of single variables was between -0.48 and 0.44.

## Conclusion

Adaptation of the elastic net to canonical correlation analysis considerably reduces the number of variables within each CCA component without too much loss of information, and thus makes the interpretation of the CCA components easier.

The penalized CCA deals well with situations where the number of variables greatly exceeds the number of subjects. Furthermore, strong intraset correlation is accounted for by the grouping effect of the ridge penalty parameter. An additional advantage is that the penalized canonical correlation analysis has a built-in variable selection procedure, so multiple testing is much less problematic.

In this paper we only focussed on the first set of CCA components, even though the canonical correlation of the second set of CCA components is quite high. For example a residual matrix from a first pair of CCA components containing 200 variables each had a canonical correlation of 0.70 for a pair of CCA components with 50 variables each. Further analysis seems fruitful.

## Competing interests

The author(s) declare that they have no competing interests.
